# Postpartum Care Recommendations from Parents of Premature Infants Requiring Intensive Care

**DOI:** 10.1007/s10995-025-04101-x

**Published:** 2025-04-28

**Authors:** Melissa J. Chen, Laura R. Kair, Eleanor Bimla Schwarz, Melissa Toland, Julianne Rizzo, Mitchell D. Creinin, Judy C. Chang

**Affiliations:** 1 Department of Obstetrics and Gynecology, University of California, Davis, 4860 Y Street, Sacramento, CA 95817 USA; 2 Department of Pediatrics, University of California, Davis, 2516 Stockton Blvd, Sacramento, CA 95817 USA; 3 Department of Medicine, Division of General Internal Medicine, University of California, San Francisco, 1001 Potrero Ave. Pride Hall, San Francisco, CA USA; 4 University of California, Davis School of Medicine; Current Affiliation with Department of Obstetrics and Gynecology, Harbor-UCLA Medical Center, West Carson, CA USA; 5 University of California, Davis School of Medicine; Current Affiliation with Department of Internal Medicine, University of California, Irvine, CA USA; 6 Departments of Obstetrics, Gynecology & Reproductive Sciences and Internal Medicine, University of Pittsburgh, 3240 Craft Place, Suite 229, Pittsburgh, PA 15213 USA

**Keywords:** Postpartum period, Qualitative research, Healthcare services, Neonatal intensive care unit, Maternal health

## Abstract

**Objective:**

To describe postpartum care preferences and experiences among individuals who deliver a premature infant requiring neonatal intensive care.

**Methods:**

In this qualitative description study, we recruited patients 2 to 8 weeks after delivery of a premature infant requiring neonatal intensive care to participate in semi-structured interviews. We asked participants to share their postpartum care experiences including their expectations and preferences regarding what is addressed during postpartum visits, their decision-making process in attending scheduled postpartum visits, and their suggestions for how to optimize postpartum care to serve their needs. We used thematic analysis to generate codes and identify themes.

**Results:**

Of 26 participants, 8 (31%) had attended a postpartum visit, 4 (15%) had missed their appointment, and 14 (54%) had a visit scheduled to occur after the time of the study interview. We found that participants weigh the perceived benefits of attending a postpartum visit against barriers to care, such as insurance restrictions, competing responsibilities and priorities when deciding whether to attend their postpartum visit. At their postpartum visit, participants preferred when clinicians centered the visits around the participants’ goals and tailored the encounter to their specific concerns. Lastly, participants recognize that screening for postpartum mood disorders is important; however, the current screening tools do not differentiate between mood disorders and expected responses to a stressful neonatal intensive care experience.

**Conclusions for Practice:**

Postpartum visits tailored to patient preferences for care in the early postpartum period are needed alongside system-level interventions to address barriers to accessing postpartum care for patients who deliver premature infants.

**Supplementary Information:**

The online version contains supplementary material available at 10.1007/s10995-025-04101-x.

## Significance

What is already known on this subject?

What this study adds?

Despite the potential benefits of promptly accessing healthcare after delivery, women with premature infants requiring neonatal intensive care often delay and receive inadequate postpartum care.

Patient perspectives should guide tailoring of postpartum in the early postpartum period and system-level interventions to address barriers to care for women with premature infants who are hospitalized.

**Supplementary Information:**

The online version contains supplementary material available at 10.1007/s10995-025-04101-x.

## Introduction

The first 12 weeks postpartum, also known as the “fourth trimester,” is an important time to address concerns including physical recovery from childbirth, maternal mental health issues, infant feeding, chronic disease management, and health maintenance (ACOG, 2018; Tully, 2017). Individuals with premature infants requiring hospitalization in a Neonatal Intensive Care Unit (NICU) have a higher burden of chronic disease and are more likely to experience complications such as hemorrhage and infections (Reddy, 2015; Verbiest, 2020). In addition, they are more likely to develop postpartum depression, acute stress disorder, post-traumatic stress, and anxiety because of the traumatic birthing experience (Lefkowitz, 2010; Beck, 2017).

Despite the potential benefits of promptly accessing healthcare services after delivery, parents of premature infants requiring NICU hospitalization are often at risk for delayed or inadequate postpartum care; one potential reason is that people with infants in the NICU often prioritize the needs of their infant above their own (Verbiest, 2020; Ferrari, 2022). Understanding the individualized postpartum needs and visit experiences of those with infants in the NICU may help optimize maternal health, infant care, and future birth outcomes. We conducted a qualitative study using semi-structured interviews to better understand reproductive health experiences, needs, and decision-making among patients with premature infants in the NICU. In this analysis, we aimed to understand the perceptions of postpartum care needs and experiences of these patients.

## Methods

We recruited individuals who delivered a premature infant requiring neonatal intensive care at our institution’s NICU to participate in a qualitative study using semi-structured interviews about their pregnancy, delivery, NICU, and postpartum care experiences. This level IV nursery serves families throughout Northern and Central California, including those who had received prenatal care primarily at the institution, those who transferred obstetric care to deliver a high-risk pregnancy at the institution, and those who delivered at another hospital and whose infants were transferred to this facility for high-level neonatal care.

Our qualitative study methods have been previously described in detail (Chen et al., 2022). Briefly, eligibility criteria included age 15 to 45 years, singleton delivery prior to 37 weeks’ gestation with the infant requiring NICU admission, and English-speaking. Those who had infants considered unstable or critical by NICU staff were excluded. We recruited participants using posted advertisements and healthcare provider referrals. Among eligible participants who responded with interest, we used purposeful sampling to ensure a heterogenous sample with respect to age, race and ethnicity, and parity. During the consent process, the principal investigator informed eligible participants that she was a doctor at the hospital who was interested in understanding how to better support patients who delivered prematurely. The principal investigator did not have a prior relationship with any of the study participants. The principal investigator conducted all the interviews after participants provided informed consent. The principal investigator is an Asian female obstetrician/gynecologist with subspecialty training in complex family planning. Her practice includes patients seeking postpartum contraception. Individual interviews took place at 2 through 8 weeks after delivery, the timeframe during which most patients access postpartum health services, in a private space in the NICU or over the phone. No other individuals were present during the interviews The interview included questions about preferences for and experiences with postpartum care, including what topics the participant felt were important to discuss with the doctor at their postpartum visit, the aspects participants liked or would change about the postpartum visit, reasons for not attending a postpartum visit if they missed their appointment, and what the participants would want to talk about if the doctor asked how they were feeling in relation to postpartum depression (Appendix). The interview guide was pilot tested, and no repeat interviews were conducted. Participants received a $50 gift card upon interview completion. The University of California, Davis Institutional Review Board approved this study.

We used a qualitative description design to develop an understanding of postpartum care among individuals with premature infants requiring intensive care through gathering descriptions of the who, what, and where of this experience from participants. With this approach, we aimed to learn from the participants’ experiences to guide recommendations for clinicians (Bradshaw, 2017; Sandelowski, 2000). Interviews were audio-recorded and transcribed verbatim. Two investigators (a female obstetrician/gynecologist and a female pediatrician) reviewed and coded all transcripts using an editing organizing style for qualitative analysis by developing and updating the codes as the interviews progressed (Crabtree, 2022). During this first pass of coding, any mention of postpartum care was noted. For this analysis, two research team members (both female fourth-year medical students) reviewed all the transcripts related to postpartum care and further refined the coding for a more detailed and deeper analysis. Participants were enrolled until thematic saturation was achieved, meaning we no longer identified any additional themes related to the primary goal of the study, which occurred after 26 interviews. The team then met again to identify patterns, categories, and themes from the codes, review and refine themes, and define the final themes related to postpartum care (Vaismoradi, 2013). We did not check back with study participants to provide feedback on the findings. We used NVivo 12 software (QSR International) for organizing codes and creating the codebook and followed COREQ criteria for reporting this study.

## Results

### Participant Characteristics

We recruited 26 participants from January 2018 through February 2019. Obstetric and demographic characteristics of participants, by postpartum visit attendance, are shown in Table 1. Twelve had previously given birth. Overall, 10 (38%) participants received prenatal care and delivered at the study site, 6 (23%) received care at another clinic or hospital system and were transferred to the study site for delivery, and 10 (38%) had their infants transferred to the study site for intensive care after delivery at another hospital. Most participants (23, 88%) were staying within 30 min of the NICU, either at home or at a temporary housing facility for families of hospitalized individuals. The remaining three participants commuted between 30 min to an hour from their home to the NICU.

**Table 1:** Demographic and obstetric characteristics of participants by postpartum visit attendance during neonatal intensive care (*N* = 26)

Characteristic	Attended postpartum visit (*n* = 8)	Did not attend postpartum visit (*n* = 4)	Has upcoming postpartum visit scheduled (*n* = 14)
Age (years)	31 (29–41)	32 (26–40)	28 (18–37)
Public insurance	3 (38)	3 (75)	9 (64)
Delivered outside of study site	3 (38)	1 (25)	6 (43)
Housing while infant hospitalized			
Temporary housing near hospital Home within 30 min of hospital Home more than 30 min from hospital	2 (25) 4 (50) 2 (25)	2 (50) 2 (50) 0	8 (57) 5 (36) 1 (7)
Multiparous	3 (38)	2 (50)	7 (50)
Cesarean delivery	8 (100)	2 (50)	7 (50)
Timing of study interview (days postpartum)	39 (25–55)	23 (16–49)	19 (14–40)

Note: Data described as median (range) or n (%)

Interviews were completed at a median of 39 days (range 25–55 days) after delivery, and the median interview duration was 35 min (range 22–62 min). Eight (31%) participants had attended a postpartum visit; four (15%) were not planning to attend a postpartum visit due to insurance changes or had missed their appointment. The remaining 14 (54%) had an upcoming appointment scheduled; these participants, were interviewed at a median of 19 days postpartum (range 14–40 days).

We identified three key themes from participants’ interviews: participants weighed the perceived benefits of going to a postpartum visit against the challenges of overcoming numerous barriers to make it to these visits; participants perceived postpartum visits to be helpful and positive experiences when their needs and concerns were prioritized; and postpartum mental health assessments need to consider the context of having a NICU infant.

### Weighing the Benefits of and Challenges with Attending a Postpartum Visit

Participants all had medical concerns related to their pregnancy, delivery, or postpartum recovery process to discuss with their physician, such as assessment of physical recovery after birth, counseling about nutrition after childbirth, assessment of postpartum mood, review of pregnancy events, future pregnancy planning, and breastmilk feeding and infant care (Table 2). However, each individual weighed the perceived value of receiving postpartum care against any barriers to attending the postpartum visit. Those who did not attend their postpartum visit cited barriers including insurance changes and prioritization of the infant’s health over their own. The time and energy associated with addressing these barriers outweighed the potential benefits of the visit, especially if contraception initiation was not a concern. For instance, one participant whose insurance changed during her pregnancy stated:

**Table 2:** Desired topics of discussion at the postpartum visit with representative quote by participants with premature infants requiring intensive care

Topic	Representative Quote
Physical recovery after childbirth	Mostly, right now, I want to make sure, because I had an emergency C-section, that my incision is not infected and that I’m healthy so I can take care of my baby. – Participant 2, upcoming postpartum visit, Cesarean delivery at 28–32 weeks
Postpartum mood	Everyone kept on saying before I was discharged from the hospital that I was at really high risk for postpartum depression, even more so just because I did have a preterm delivery. I don’t know what that feels like cause I feel like anybody in this situation would be overwhelmed, or have anxiety, or at some point, not be able to sleep, or not be able to eat, and I don’t know the difference between postpartum and what I’m dealing with. So that’s something I would probably have more questions about. - Participant 3, Did not attend postpartum visit, vaginal delivery at < 28 weeks
Nutrition after childbirth	What should I be eating because I’m always, like, what should I eat to heal or, I don’t know what, get back, back on track… Just maybe not getting enough iron because you lose lots of blood, so, the iron and, I mean, I do eat healthy, but I mean, he might, they might know better what else is important or, like, what – if there’s any vitamins probably just I should be looking into. – Participant 12, upcoming visit, vaginal delivery at < 28 weeks
Review of pregnancy events	I definitely want to ask her why she thinks I went into preterm labor…And then the day before I went into labor, I did a yoga class. But I’ve been doing yoga the whole time. So, all the OBs in Labor and Delivery were like, “No, we don’t know why; that’s not why.” But I kind of want to see what she has to say because she knows my whole medical history. – Participant 17, upcoming appointment, vaginal delivery at 32–36 weeks
Discussion of future pregnancy planning	I would be curious to know if I get pregnant again, what are the chances of the baby coming early or earlier. Because that would be a real concern, because I want to have another kid, but if there’s a high chance of the baby being born early because of my first pregnancy that came early, that would be a concern. – Participant 8, upcoming appointment, vaginal delivery at 32–36 weeks
Advice about breastmilk feeding and infant care	Well, I’m definitely having some issues breastfeeding. So, I was, I would definitely want to ask some questions about that. Mainly because, breastfeeding I mean, especially because she’s a preemie as it is, like its super important. – Participant 22, upcoming postpartum visit, vaginal delivery at < 28 weeks

Because of all my emotions and not knowing what is what, I do feel like it’s very important (to see the doctor after delivery). It would have been a lot easier if I wasn’t dealing with insurance issues… I don’t really have the energy or the effort to put in, right now, to try to find – to establish with a doctor and to take care of my postpartum visit with a medical group who doesn’t know my history or doesn’t know me for that…No, just because I’m under so much stress now. I probably wouldn’t even think twice about having sex again, and I think that’s, like, the biggest concern is not having a baby, the reason why you have birth control. So, it’s not that big of a deal to me that I’m not having that postpartum visit based on the birth control aspect. – Participant 3, Did not attend postpartum visit, vaginal delivery at < 28 weeks.

Participants who had been transferred to the study site, either for delivery or after delivery, faced additional logistical challenges especially if their obstetrician was not located near the infant’s hospital. These challenges, which included finding transportation and taking time away from being at their infant’s bedside to travel, were taken into consideration when deciding whether to attend a postpartum visit. For participants who were transferred to the study site for delivery, some expressed a preference to have their postpartum visit with their delivery physicians because of convenience and continuity of care but were unable to do so due to insurance barriers. Consequently, participants either did not seek care or accessed services through the inpatient labor and delivery unit if needed. As one participant summarized when asked about their reasons for not attending the postpartum visit:

A. I’d never had an OB here. B, I don’t care about what’s going on with me right now, I only care about what’s going on with (baby) and my other daughter. Like, main focus. I know my tubes are taken out, I stopped bleeding, my scar’s healed, like I can manage with the meds that I have, even though I’m struggling. It is what is it is right now. – Participant 4, did not attend postpartum visit, Cesarean delivery at 32–36 weeks.

### Individualizing Postpartum Care

For those who attended a postpartum visit, certain aspects of their visit produced a more positive experience, such as when the clinician created a safe space for asking questions and did not make the participants feel rushed for time. Participants also remarked that they preferred when they were able to guide the conversation with topics that were important to them, rather than feeling like the clinicians were just going through a checklist. In contrast, those who found their visit less valuable commented on feeling like the visits were too short or impersonal. As one person shared:

I feel like it was a very quick and just kind of, like, you know, I’m sure she had other patients, so it was, like, kind of passing though. It wasn’t really more of, like, a sit down, let’s discuss… Like the whole asking about when if I planned on having more kids or, like, and when. I guess that would have been really nice to discuss because mostly I think we just discussed how my incision was looking and, you know, if my uterus was back to its normal state, and basically that’s it. – Participant 5, attended the postpartum visit, Cesarean delivery at 28–32 weeks.

Participants also noted their preferences to individualize the timing of the postpartum visit. Some participants who had a visit scheduled at 4–6 weeks after delivery would have preferred an earlier appointment to address initial concerns and then schedule a follow-up visit if needed. One participant with an upcoming visit at 4 weeks postpartum indicated:

I kind of think maybe four weeks is a little far out. I don’t know. I think maybe two weeks would have been better, just to kind of have your body checked. And like I have a couple of questions… Just because it seems like what if something was going on and I have to call and basically try to get fit in. You know? I think it would be better for a doctor to see each mom sooner. Just because they can assess – because every case is different. – Participant 11, upcoming postpartum visit, vaginal delivery at 32–36 weeks.

### Postpartum Mood Assessment in the Context of Infant NICU Hospitalization

When asked about their feelings related to identifying postpartum depression, participants described wanting to discuss how to differentiate between an expected response to the stress of having a NICU infant with postpartum blues, depression, or anxiety, especially after being told that they were at higher risk of having postpartum mood disorders. As one participant said:

Because I know when I was discharged here she said like, “You know, there’s the postpartum blues and then there’s the postpartum depression.” So, I guess maybe like what would be like a major difference? Okay, now, I’m transitioning from blues to depression. Because I mean I feel like I’m mentally okay. Of course, this has all been very difficult, but I don’t think I’m drifting into that state yet. So, I just hope like I know my own warning sign… So, maybe having more examples I guess. – Participant 9, Cesarean delivery at < 28 weeks.

Participants who had attended a postpartum visit and undergone screening for postpartum depression with a survey remarked on the low utility of the tool for identifying depression given the overlap of symptoms of mood disorders and their stressful circumstances. As one participant elaborated:

We talked a little bit about the postpartum depression survey…that I felt like I understand that postpartum depression is a big deal and very prevalent and important to identify, but that it doesn’t really work for some women in the NICU experience. Because the questions are like, you know, have you cried more frequently? Yeah. Do you feel sad often? Yeah, so differentiating between being depressed and sort of having reasonable and appropriate emotions for the situation isn’t really there. – Participant 24, attended postpartum visit, Cesarean delivery at < 28 weeks.

Instead of relying on the survey tool to identify postpartum depression, participants reflected on ways they were differentiating between an overwhelming experience of having a preterm infant in the NICU and postpartum depression for themselves. One participant brought up whether they could still find joy in other aspects of life at that time and another considered whether the depressed feelings persisted or were situational based on how their children were doing in the NICU.

And watching my daughter every day, and she struggles some days, and she had bad days. Like it makes me super sad. And it makes me feel a little depressed. But it’s not something that sticks, does that make sense? So, when I go home, you know, or I come back the next day and I see, oh my God, she’s doing so much better. Like it’s a totally different situation. And I don’t feel like I’m depressed all the time. – Participant 22, upcoming postpartum visit, vaginal delivery at < 28 weeks.

## Discussion

In this qualitative study, we found that people who gave birth to a premature infant requiring NICU hospitalization weigh multiple considerations when deciding whether to seek postpartum care. Participants identified clinic and systems-level barriers to care, including provider availability and insurance restrictions, that particularly impact patients requiring transfer to tertiary or quaternary hospitals for care. While factors such as the urgency of the medical concern can tip the balance towards obtaining care, encountering these barriers can lead to the decision to delay or even forgo postpartum care, putting these patients at risk for inadequate or incomplete treatment of their medical conditions. These findings have also been described in prior studies in which patients with infants in the NICU will prioritize their infants’ medical care above their own health needs after delivery (Verbiest, 2020; Ferrari, 2022). Interventions to streamline access to care, such as co-location of postpartum care in the hospital/NICU or insurance companies automatically covering postpartum care at the transferring facility when a NICU admission occurs could potentially address these systems-level barriers (Verbiest, 2016; Caskey, 2021; Polk, 2021; Tan, 2023; Glassgow, 2023; Handler, 2021).

In this study, participants also identified that current postpartum screening tools may not be relevant when neonatal intensive care is required. Previous data indicate that women with premature infants in the NICU are at high risk of experiencing depressive symptoms (Garfield, 2021; Levinson, 2022), and screening for postpartum mood disorders at postpartum visits is important to identify those who would benefit from treatment (ACOG, 2018). However, commonly used screening tools, such as the Edinburgh Postnatal Depression Scale, have yet to be validated among parents of NICU infants (Cox, 1987). Therefore, using the same threshold for a positive screen may not be appropriate with patients who are experiencing significant stressors related to their infant’s hospitalization. Until additional research clarifies ideal approaches to screening parents of infants requiring intensive care, clinicians should frame conversations responding to screening results with statements about normal responses to stressful situations and relevant resources that are available during the postpartum period.

Lastly, our findings suggest that the postpartum visit goals were misaligned between some participants and their clinicians, and clinicians who individualize postpartum care may improve engagement in care by increasing the perceived importance of the postpartum visit from the patient’s perspective (Tierney, 2024). Participants described communication practices that improved their postpartum appointment experience, such as when clinicians made time to address the patients’ concerns instead of checking off a list of prespecified items during the visit. Notably, these patient-centered practices are not unique to providing postpartum care for patients with premature infants requiring hospitalization. Further, appointments that devalue patients’ experiences may deter patients from seeking future postpartum care as seen when participants report “no need for it” as a reason for not receiving postpartum care (DiBari, 2014).

One strength of this study is the inclusion of participants with a variety of perspectives of expectations and experiences of postpartum care. However, we only recruited English-speaking individuals from a single site, which limits the generalizability of these findings. This study was also performed prior to the COVID-19 pandemic, and postpartum care experiences may be different now with the increased use of telehealth after the pandemic. Even though the principal investigator did not have any prior relationships with the study participants, participants may have been affected by social desirability bias as the principal investigator was a physician at the study institution and the sole interviewer for the study. Lastly, we recognize that recruitment occurred early in the postpartum period (2–8 weeks) and that many of participants had a scheduled postpartum visit after the time of the interview, which may have affected our findings. Further, additional themes may have emerged with recruitment of participants later in the postpartum period (e.g., after 8 weeks postpartum).

In summary, patients with premature infants in the NICU have complicated experiences with pregnancy, delivery, and newborn care that require tailoring postpartum visits to meet their personalized needs. Ongoing efforts by healthcare systems to address patient, clinic, hospital, and reimbursement-related barriers are needed to facilitate timely receipt of high quality postpartum care.

## Electronic Supplementary Material

Below is the link to the electronic supplementary material.

Supplementary Material 1

## Data Availability

With approval from investigators.
