# Supplementary material for: Postpartum Care Recommendations from Parents of Premature Infants Requiring Intensive Care
Source: Matern Child Health J. 2025 Apr 28;29(6):818–24. doi: 10.1007/s10995-025-04101-x (PMC12206205; doi:10.1007/s10995-025-04101-x)
Supplement: Supplementary file 1 — Supplementary Material 1 [file 10995_2025_4101_MOESM1_ESM.docx]

Appendix 1: Interview guide

Domain: Pregnancy and NICU experience

- Could you start with sharing what your experience with this pregnancy and delivery was like for you?
- Can you tell me about your experience in the NICU?
  - What have you found helpful to you while your baby is in the NICU?
  - What has been particularly challenging for you?

Domain: Postpartum care

- During the postpartum period, doctors focus on certain aspects of health care to transition from pregnancy to routine care. What do you think is important for you to discuss with your doctor at the postpartum visit?
  - If attended postpartum visit: what did you like about the visit? What would you want to change?
  - If did not attend postpartum visit: what were some reasons why you didn’t go to the visit?
- One aspect that doctors focus on is checking for postpartum depression. If a doctor asked you about how you are feeling, what would you want to talk about?

Domain: Contraception counseling thoughts and experiences

- Another aspect of health care that doctors talk about after delivery is birth control. For you, what has your experience been regarding talking about birth control since the birth of your baby?
  - How do you feel about starting to have sex again after delivery?
  - When, if ever, are you thinking you might want to become pregnant again?
  - Can you tell me who you’ve talked to about birth control after your delivery?
  - How did that conversation go?
  - What did you like about it?
  - What would you have preferred instead?

Domain: Contraceptive preferences postpartum

- What are your thoughts about starting a birth control method after delivery?
- What did you like or not like about the methods you have used in the past?
- Which option are you thinking about using/did you choose?
- What are some factors that are important to you when choosing a birth control method?
  - How does your health history affect your birth control decision?
- What are your thoughts about whether or not your birth control method could affect your breast milk supply?
- How do you think having a preterm delivery affects your decision about which birth control method to use?
- How does having a preterm delivery affect your thoughts about having more children in the future?

Domain: Infant feeding goals

- Another aspect that doctors focus on after delivery is to help women with feeding their infants. We know that premature babies can get nutrition in many different ways, and I’ll refer to this as “infant feeding”. What has been your experience with feeding your baby?
  - Before the delivery, what had been your thoughts and plans about feeding your baby?
  - What has been your previous experience with infant feeding?
  - What was important to you as you thought about how you would feed your baby?
    - Were you thinking about breastfeeding?
    - (If yes) How do you feel about breastfeeding now?
    - How does your health history affect your decision to breastfeed?
- Tell me about how feeding your baby has gone?
- What has your support been like?
- What are your thoughts about feeding your baby after you leave the hospital?

Is there anything else I should have asked you or talked about that you’d like to share?
